# Frequency and Content of the Last Fifty Years of Papers on Aristotle’s Writings on Biological Phenomena

**DOI:** 10.1007/s10739-022-09683-8

**Published:** 2022-06-29

**Authors:** Christopher F. Sharpley, Clemens Koehn

**Affiliations:** 1 Brain-Behaviour Research Group, School of Science & Technology, University of New England, Armidale, NSW 2350 Australia; 2 Department of Archaeology, Classics and History, University of New England, Armidale, NSW 2350 Australia

**Keywords:** Aristotle, Biology, Citation analysis, Digital humanities

## Abstract

Aristotle is often named as the first zoologist or biologist because of his writings on animals. Although Aristotle’s major intention in these books was to illustrate his ideas of how knowledge and understanding might advance, at least one modern biologist (C. Darwin) has recognized Aristotle's depth and breadth as being of surviving merit. Of greater surprise is the ongoing attention that his works continue to receive, including publications in contemporary scientific journals. This review identifies 38 peer-reviewed papers on various topics from Aristotle’s biological writings that have been published during the last 50 years. These papers are described according to content (genetics, population biology, anatomy, brain, movement), specific creatures (fish, scorpions, elephants, insects, birds), publication outlet, distribution over the fifty year period surveyed, and visible trends in the topics studied. It is concluded that, in the highly-competitive field of peer-reviewed scientific publication and citation, Aristotle's biology continues to excite the interest of scientists and remains salient to modern science itself.

## Aristotle and Modern Science

The relevance of Darwin's comment (above) to his friend William Ogle lies in the achievements of Darwin's two "gods." Linnaeus developed modern methods of classifying animals and plants (taxonomy) (Herbaria 2019). Cuvier extended Linnaeus's ideas by classifying organisms into *phyla* that included fossils as well as living species (Faria, 2010), work that was admired by Darwin (Gotthelf 1999).^1^ When he compares Aristotle with Cuvier and Linnaeus, Darwin clearly considers Aristotle to have made a greater contribution to modern science and biology in particular. To elucidate that contribution as it continues today, this paper attempts to (1) identify more recent scientific publications about Aristotle’s works on biological phenomena, (2) discuss the major foci of those papers, and (3) demonstrate how Aristotle continues to contribute to current scientific thinking on biology.^2^ The decision to focus on these scientific works does not deny the value of other, more philosophical papers that discuss the metaphysics of Aristotle’s biology, but rather it augments those papers by examining what is being written about a historical event in biology (that is, Aristotle’s writings) from a scientific perspective and for modern students of biology.

Darwin's comment about Aristotle was made in 1882. More recently, there has been a renewal of interest in what might (from a contemporary perspective) be called Aristotle’s biological writings in the last 50 years or so by two sets of authors with distinct motivations (Tipton 2006, 2014). First, there are biologists (some eminent) who have been impressed by the rigor of Aristotle’s observations and want to bring these to the attention of modern scientists; second, there are philosophers and classicists who seek to find out how Aristotle’s writings on biology fit into his other works on metaphysics, ethics, and physics.

Although Tipton (2006) claims that there has been renewed interest in Aristotle’s biology, he does not itemize that interest by listing and describing the papers published, any trends in frequency over time, or the foci of those papers. Such an examination of the published works on Aristotle’s biology has the potential to provide a sense of perspective to the history of biology in terms of these aspects and also to generate some hypotheses regarding the possible reasons why interest in Aristotle’s biology has been revived.

This paper is concerned with only the first group of publications mentioned above (that is, those directed towards readers of zoological papers, albeit from a historical perspective) in order to ascertain how Aristotle’s biology is being made relevant to twenty-first-century biologists and to students of the history of biology.^3^ Such a focus is not patronizing on the scientific side; on the contrary, it deliberately reflects on the trend in the other areas concerned with Aristotelian studies: philosophy and classics. These other areas, rather than seeing Aristotle as a philosopher only, increasingly stress the enormous impact Aristotle’s biology has on the other topics that Aristotle writes about (ethics, physics, philosophy, etc.). Thus, there is a correlation detectable between the renewed interest in how Aristotle’s biological ideas contribute to modern science and the opening up of the other areas for a more science-focused discourse, whether it be Aristotle’s social and political writings (Horowitz 1976; Kullmann 1980), philosophical works (Gotthelf 2012; Tipton 2014), or linguistic studies (Laspia 2018).

While the influence that his work on biology had on Aristotle’s overall research program (when acknowledged) has, in previous decades, often been interpreted in a dichotomous pattern (as in the case of Aristotle’s understanding of gender, usually in a misogynistically-assumed interpretation; see Horowitz 1976), recent scholarship is more cautious and follows a more-encompassing pattern of explanation rather than quickly ideologizing its findings (for example, on Aristotelian gender views, see Mayhew 2004). Therefore, while not arguing that the renewed interest in Aristotle’s biology is only a matter relevant for modern biologists, we concentrate here on scientific papers *strictu *sensu because the influence and intensity of discussion of this work on the side of the sciences is still greater than in classics and philosophy.

From a cross-disciplinary perspective, this momentum of the relevance that Aristotle is currently regaining in modern science should continuously alert humanities scholars to the significance of Aristotle’s biological works for the overall assessment of his intellectual personality and life achievement. Although this primary focus upon the relevance of Aristotle’s biology for modern sciences might be interpreted as 'politicizing' his work in favor of scientists, that focus also highlights one legitimate, if present-focused, history of biology that demonstrates how Aristotle’s work bridges a gap of some 2300 years and remains relevant to twenty-first-century science (Horowitz 1976).

## Literature Search Strategy

Five strategies were followed to identify recent research papers that were (1) focused upon the scientific relevance of Aristotle’s biology, (2) not concerned with philosophical aspects of his model of scientific inquiry, and (3) truly aimed at bringing the value of Aristotle’s biology to the attention of the modern reader in science and in the history of biology itself. First (as recommended in the findings reported by Martín-Martín et al. [2018]), a search was conducted in Google Scholar on May 3, 2020 using the descriptors “Aristotle,” “biology,” “animals,” and “modern science,” chosen after a search of citations used in ten papers that we had already identified and whose descriptors we then adopted.^4^ This method produced about 37,000 results for the fifty years from 1970 to 2019, sorted by relevance.^5^ Of these results, the first 500 peer-reviewed journal articles were selected and their abstracts read for relevant content.^6^ Second, the reference lists of each paper were manually searched for relevant articles. Third, a citation search in Google Scholar for each of those articles (that is, subsequent papers that had referenced them) was also undertaken.^7^ Fourth, the bibliography from the specialized 2014 text by Leroi (2014) about Aristotle’s biological research on Lesbos was examined. The comprehensive *Oxford Handbook of Aristotle* (Shields 2012) also provided additional sources. Finally, the search descriptors were entered into PubMed and Science Direct to identify any papers that did not appear in Google Scholar.^8^

These five processes produced a total of thirty-eight papers for inclusion in this review. As such, the selection of papers is appropriately narrow. To tie these papers to Aristotle’s writing, quotes and page references are given (where possible) for examples, using the Work title, Book number, and Bekkeri line notation, which denotes the columns as ^a^ or ^b^ (first or second columns), page numbers, and line numbers (Bekkeri 1831).

## Search Findings

### Cursory Comments

Several papers make only brief or cursory comments upon Aristotle’s biology. For example, Grene (2000) notes the state of current scientific debate regarding Aristotle’s supposed *genetics*; de Magalhaes and Costa (2009) cite Aristotle’s descriptions of the diversity of lifespans observed in nature; Brussow (2009, p. 2263) reports that Aristotle’s books offer “careful descriptions, sharp reasoning, the beginning of experimentation and entertaining errors;” Auer et al. (2007) refer to Aristotle’s development of comparative anatomy via dissection of animals; and Autumn et al. (2014) remind us that Aristotle observed that the gecko “could run up and down a tree in any way, even with its head downwards” (Aristotle, *History of Animals*, IX, 614^b^ 2–3). Föllinger (2012) comments on the use by Aristotle of “dialogical elements,” and many papers used Aristotle’s biology in an introductory manner, briefly mentioning his work and then passing on to a non-related discussion of more modern findings (see Sodergren et al. 2006) or a review of historical processes (see Carlos et al. 2019).

### Analytic Process Papers

Some authors focus on the analytic processes used by Aristotle to classify animals and generally conclude that there is “a fairly consistent underlying classification in the zoological works of Aristotle” (von Lieven and Humar 2008, p. 227). Similarly, it is strongly argued by Grene (1972, p. 423) that Aristotle’s biology reminds us of the “many-levelled structure of biological systems” and, hence, the “inadequacy of a one-levelled atomism” that sometimes overtakes modern biology in the form of reductionism to physics and chemistry (Grene 1972, p. 395). In dealing with what he refers to as the "genus-as-matter/species-as-differentia" account of the genus-species relation found in the *Metaphysics*, Lennox (1980, p. 324) argues that Aristotle introduces the constructs of “the more and the less” to differentiate between different species within the same genus in *Parts of Animals.*^9^ Lennox admits that this distinction would not stand against modern definitions of genus and species, although this opinion is not shared by all. Pellegrin, for example, argues that “the two terms seem to have, at least approximately, the significance of ‘genus’ and ‘species’ in the modern sense” (Pellegrin 1986, p. 76). In his discussion of the Triune Brain as described by Plato, Aristotle, and Erasistratus, Smith (2010) sets out each account of the three types of “soul” and shows that Aristotle was the only one of these three ancient philosophers to argue that the soul was within the body.^10^ Corsilius and Gregoric present a model for resolving the apparent contradiction between Aristotle’s claims that “the soul is the efficient cause of animal motion” as well as “being the internal supporting-point necessary for animal motion” (Corsilius and Gregoric 2013, p. 52).

Although these papers focus upon more general aspects of Aristotle’s writing on biology, they are relevant to the papers on specific topics (described below) because they cover what might loosely be termed the *scientific method* as Aristotle was developing it. As such, they reflect his overall approach to (for example) general problems like speciation and classification. They also link Aristotle’s biological works to the rest of his writing.^11^ However, the question addressed by this paper may be answered in more detail by reference to peer-reviewed publications about *specific topics* in Aristotle’s biology. Several key areas in which such papers have been written are summarized below to provide a flavor of the kind of writing about Aristotle’s biology that has occurred during the last fifty years of relevant scientific literature.

## Specific Topics

### Genetics

Although it has been suggested that Aristotle all but set down a model of gene-based inheritance (Delbrück 1971), that view is strongly challenged as overly simplistic and mechanistic and attributing to DNA a much more controlling role by “posit[ing] a decidedly un-Aristotelian genetic vitalism” (Vinci and Robert 2005, p. 221). One of the most influential ideas in modern biology is *epigenetics*, first coined by Waddington and defined as “derived from the Greek word *epigenesis*, which Aristotle used for the theory that development is brought about through a series of causal interactions between the various parts” (Waddington 1956, p. 10). While no argument is being made here that Aristotle was responsible for the entire modern concept of epigenetics, Waddington considered that the *Parts of Animals* contributed to the development of that concept.

Further comment on Aristotle’s contributions to genetics comes from Kullmann, who sets himself “to point out some striking examples which show that Aristotle's thoughts may yet play a remarkable role in contemporary scientific discussion” (Kullman 1991, p. 137). Although most of his paper concentrates on Aristotle’s overall contributions,^12^ such as his (unintended) taxonomy,^13^ Kullmann makes a case for Aristotle as a proto-geneticist, even arguing that Aristotle took this role in ways that were superior to Darwin’s model^14^ because Aristotle argued that (in Kullmann’s words) “the purposeful structure of a living being depends on the programme pre-existing in the blood of the parents” (Kullman 1991, p. 146).^15^

Henry discusses the question, What are the causal mechanisms behind the transmission of biological form? (Henry 2006, p. 426). He argues that the answer lies in the *Generation of Animals*, Book 4, where Aristotle describes the phenomenon of inheritance.^16^ Aristotle maintains that there are a set of “movements”^17^ that transmit the parents’ form to their offspring and that these movements “exist in the semen; potentially from remoter ancestors but in a higher degree from whatever individual is nearer.”^18^

### Population Biology

The purview of population biology includes “age structure,” and Egerton (1975, p. 307) considers that “almost all” of Aristotle’s biology was relevant to this construct. Egerton also argues that *Aristotle’s History of Animals* encompasses age structure in great detail, and uses the examples of mammals and birds to illustrate how Aristotle does this.^19^

### On Animals and Humans

Perhaps due to his experiences as a child (Shields 2012),^20^ Aristotle developed a good understanding of native wildlife, exotic species, and farmed animals (Grumett 2019), and he devoted quite a lot of time to describing the care of farm animals.^21^ Because he considered that animals have a soul similar to humans, and that animals’ *telos* is to confer their attributes and products onto their owners (for example, a cow’s milk, a sheep’s wool, a chicken’s eggs), then “a stockperson’s primary role is to promote the good of the animals in their care, ensuring that their needs, wants and desires are satisfied.”^22^,^23^ Aristotle gave many examples of how this may be accomplished.^24^

Aristotle asserted that, if a hen bird has mated with a cock and is pregnant, then if “she be trodden by another cock, the whole brood of chicks turn out like the second cock.”^25^ Although this claim was said to be “very astonishing,” it has been verified (Birkhead and Møller 1998), and Brock notes that this is “another case where Aristotle’s empirical observations of biology, though apparently bizarre, have been vindicated by modern science” (Brock 2004, p. 278).^26^

### The Brain

Gross (1995) describes how Aristotle broke from some of his predecessors such as Alcmaeon of Croton, Democritus, Anaxagoras, and Diogenes, to state that “the brain cannot be the cause of any sensations, seeing that it is itself as utterly without feeling as any of the excretions”^27^ and that “it is the region of the heart that constitutes the sensory center.”^28^ It has been argued that this error by Aristotle arose because of his lack of clinically-focused study and that “he never dissected a human” (Gross 1995, p. 249). Aristotle lived in an era when the budding scientific methodology did not include experimentation, which forbade him from engaging in the kind of clinically-focused research that Galen undertook six centuries later that described the function of the brain and the spinal cord (Galen 1962). However, Aristotle was active in establishing the Museum at Alexandria, where dissection was undertaken, leading to the accurate description of the human brain (Longrigg 1988).^29^

Although it was published just before the search period (1970–2020), an earlier paper by Clarke and Stannard deserves mention because its authors present several arguments explaining why Aristotle reported that the brain occupied only the front of the head,^30^^,^ 31 such as Aristotle’s desire to “create a synthesis of Pre-socratic and Platonic knowledge” (Clarke and Stannard 1963, p. 132).^32^^,^ 33 Aristotle inaccurately described several human structures, including the cranial sutures, uterus, kidney, spleen, ribs, and heart, and “viewed biological phenomena from a philosophical standpoint,” with less attention to accuracy than is expected by biologists today (Clarke and Stannard 1963, p. 148).

### Anatomy

Aristotle is considered by many as “the founder of comparative anatomy,” although much of his work was based upon animals (from which he generalized to humans) because he did not perform dissections of humans (Blits 1999, p. 59).^34^ However, Malomo et al. describe Aristotle as having “laid the foundation of comparative anatomy and established embryology on a scientific foundation by his direct studies of the chick embryo” (Malomo et al. 2006, p. 100). These studies were detailed and undertaken by an ingenious method^35^ and influenced thinking in that field until well after the Renaissance.

Perhaps the strongest basis for the high opinion in which anatomists hold Aristotle is his focus upon function.^36^ Thus Blits writes: “Form and function go together for Aristotle. Anatomy and physiology are integral components of the same science” (Blits 1999, p. 62). This position is amplified by Blits when she explains that Aristotle’s task “is to discover the principles of organization and function responsible for the various animal kinds and their relations,” which entailed Aristotle in defining the differences in form between animals and the underlying "causes" why those differences occur. Aristotle identified four causes: *material*, *form*, *origin of motion* (or efficient), and *end* (or final), and in doing so, provided a model of anatomy that went well beyond a simple description of parts, but also included the reasons for why those parts are as they are and how they might differ across species. This individual variety of animals’ form and function led Aristotle to state that “each and all will reveal to us something natural and something beautiful.”^37^

In their historical article in *Clinical Anatomy* in 2007, Crivellato and Ribatti divert their discussion away from “many of his physiological concepts (that) turned out to be wrong” and instead focus on Aristotle’s development of a “precise topographical terminology” that cast the body into “a bipartite, symmetrical architecture,” including the front and back of the body, its top and what lay below, and its right and left sides (Crivellato and Ribatti 2007, pp. 477–479).^38^ Crivellato and Ribatti’s message is clear: Aristotle set down the nomenclature for most body parts and then went on to provide detailed accounts of the anatomy of the heart, blood vessels, brain, respiratory system, digestive system, kidney and urinary tract, genitals, bones and joints, muscles, and sense organs. In doing so, “Aristotle’s contribution to the development of anatomy was enormous” (Crivellato and Ribatti 2007, p. 484), and he foreshadowed the basic principle of experimentation in modern science and developed an early the concept of an organ (*organon*) as an instrumental and functional part of the body (Crivellato and Ribatti 2007).^39^ These ideas exerted “a sort of intellectual dictatorship on further generations of anatomists,” even to Renaissance thinkers (Crivellato and Ribatti 2007, p. 485) and William Harvey in the seventeenth century.^40^ Aristotle’s ideas about ways of understanding nature, plus twelfth-century translations of the wider Greco-Arabic ancient science, have been recognized as one of the three major bases of the development of the scientific revolution during the last few centuries, along with universities and the emergence of theologian-natural philosophers (Grant 1997).

In terms of the heart, Shoja et al. (2008) remind us that Aristotle reported that it consists of three chambers.^41^ In doing so, he omitted the right atrium, which he instead called a venous dilation (Shoja et al. 2008).^42^ This error has been discussed at length and is attributed to the mystical tradition attached to the number three, which is probably in accordance with Plato's notion of the three corporeal faculties (mind, emotion, desire) (Lennox 2001; Van Praagh and Van Praagh 1983). Other modern scientific papers on Aristotle’s model of the heart include Shaw’s, who notes that Aristotle made “over one hundred references to cardiac anatomy and physiology” in the *History of Animals* and *Parts of Animals* and that his “main model for the heart is simple and entirely structural: the heart is a container.” (Shaw 1972, pp. 355, 385). Shaw argues that Aristotle’s model drifted away from a more modern understanding of the heart when he attempted to describe its physiology, perhaps because of the lack of adequate laboratory settings necessary to investigate the actual way the heart worked (Shaw 1972, p. 386).

In his defense against Francis Bacon’s sixteenth-century criticisms of Aristotle’s anatomical writing,^43^ Cosans replicates Aristotle’s anatomical experiments by obtaining “anatomical material” from a Chicago meatpacking company (Cosans 1998, p. 311). This experience allowed him to understand the process used by Aristotle that “shows animals to consist of parts that are organized into wholes in virtue of dynamic forms” (Cosans 1998, p. 336). Cosans argues that, far from Bacon’s criticism, this kind of animal dissection “not only reveals the order within organisms but naturally led to Aristotle’s quest to understand the order amongst organisms” and thus contributed to the development of his more comprehensive philosophy (Cosans 1998, p. 336).

### Movement

Frampton’s paper on the role of the heart in animal movement summarizes Aristotle’s explanation of movement as follows: “The general rule of locomotion is that there must be an unmoved mover, something that is moved, and an intermediate moved mover” (Frampton 1991, p. 310). Aristotle expanded upon this simplified model by identifying that movement is initiated by “thought and desire,”^44^ and that there is “some one thing which moves” limbs, and that this is “the soul.”^45^ Although apparently despite having observed that taste and touch, sight, hearing, and smell are “lodged as a rule in the head,” for Aristotle, the soul resided in the heart.^46^

## Particular Creatures

### Fish and Marine Creatures

Tipton describes Aristotle as a philosophical biologist for whom the two disciplines were intermingled (Tipton 2006, p. 370). By focusing on two particular fish that Aristotle described (the *kobios*, which is the giant goby in modern terms; and the *phucis*, or modern intertidal benny), Tipton evaluates the accuracy of Aristotle’s descriptions of the habitat, diet, spawning, sexual dimorphism, and eggs of these fishes,^47^ which are found near Lesbos, and does so by adopting the same methods as Aristotle did (by catching these fish with hook and line).^48^ Tipton notes that his findings were mostly consistent with Aristotle’s descriptions of these two fish. However, he also notes that neither of these two fish was often eaten by Lesbos’ citizens or found in the fish markets there during Tipton’s visits to this place, suggesting that Aristotle did not completely rely on the services of information provided by fishermen but made his own observations (Tipton 2006, p. 379).

In a second paper, Tipton discusses Aristotle’s description of the feeding behavior of the red mullet (*Mullus surmuletus*), a much-fancied eating fish in Ancient Greece and still today (“in antiquity a great delicacy … and continues to be a highly-prized fish today”) (Tipton 2008, p. 166). Aristotle noted that this fish “burrows in the mud”^49^ to stir up small animals, which it then eats, which Tipton observes to be correct. Tipton also confirms that, as Aristotle noted, after the red mullet “quits its haunt, the sargue (sea bream) settles down into the place and feeds on what is left behind.”^50^

Aristotle also described the reproduction of the "dog fish," or shark,^51^ and Bodson (1983a, b) examines the statement that Aristotle is claimed to have made about these creatures’ “ovoviviparity.” Aristotle is translated as saying that “dog-fish in general can extrude and take in again their young.” Still, Bodson argues that this is not Aristotle’s intended meaning and that the “again” is misplaced. That is, while the shark is ovoviviparous, the eggs are not expelled and then taken in again by the mother. Instead, Bodson suggests that the correct translation is: “The majority of dogfish females release the young [the embryos] and keep them inside [in the uterus]” (Bodson 1983a; b, p. 403). This translation allows Aristotle’s comment about these fish to be accurate. Bodson assigns this apparent, but not actual, error by Aristotle to misinterpretation by later readers who did not attempt to assess the scientific correctness of the data.

Buddington and Diamond (1986) focus upon Aristotle’s description of “the pyloric caeca in fish,” which are blind diverticula opening “high up about the stomach.”^52^ Aristotle hypothesized these as “a sort of antechamber in which food may be stored up and undergo putrefaction and concoction.”^53^ Buddington and Diamond (1986) test this hypothesis and find that, while these caeca do not store or putrefy food, they aid in concoction (that is, digestion).

Eleni Voultsiadou and her colleagues wrote several papers about Aristotle’s descriptions of marine creatures, not all of which are relevant to this discussion, but three are of particular interest here. First, Voultsiadou et al. (2007, p. 1763) focus on the sponges of the Mediterranean, confirming Aristotle’s comments about the diversity, nature, external morphology, symbiotic relationships, habitat, and value to humankind,^54^ concluding that Aristotle’s descriptions are “more or less consistent with present scientific knowledge.” Second, her paper with Vafidis on marine invertebrate diversity in Aristotle’s biology reports on the 866 mentions he makes of marine invertebrates, including “85 current marine invertebrate taxa” (Voulsiadoun and Vafidis 2007, p. 103).^55^ By constructing an annotated catalogue of all marine *anhaima* (invertebrates) appearing in Aristotle, Voutsiadou et al.’s work is comprehensive in its tabulation and description. They conclude that “All marine animal anhaima encountered in Aristotle’s zoological works are correlated with current marine invertebrate taxa” (Voutsiadou et al. 2007, p. 114). However, there are some differences in how Aristotle classifies some animals compared to current schemes.

The third relevant paper by Ganias et al. (2017) replicates Voultsiadou and Vafidis’ (2007) paper on marine invertebrates by focusing on 109 individual fish taxa, which the authors tabulate and relate to modern classifications. Ganias et al. (2017) make the point that Linneaus used Aristotle’s fish names to construct Latinized international scientific names, although several errors in translation from ancient Greek to Latin produced confusion. In addition to this correlation between Aristotle’s and modern classification of taxa, Ganias et al. (2017) also provide examples of Aristotle’s text on fish body structure and function,^56^ reproduction and development,^57^ behavior, ecology, migration, feeding, diseases, and how they were exploited.^58^ Finally, Voultsiadou et al. (2017) reiterate their sentiments regarding the value of Aristotle’s classification schemes for the range of marine animals. They note that Aristotle also provided an account of their dispersion around the Mediterranean, including notes about water circulation patterns, bathymetry, and river sources. Aristotle also noted that fish migrated across the Mediterranean,^59^ and suggests where they could most easily be caught.^60^ Voultsiadou et al. (2017) conclude that Aristotle’s biological classification is familiar to modern marine biologists and that there are traces of his descriptions in current taxonomies.

### Scorpions

Aristotle commented that “in Pharos and other places, the bite of the scorpion is not dangerous; elsewhere—in Scythia, for instance … the sting is fatal to man or beast.”^61^ Fet et al. (2009) note that Aristotle’s comments of scorpion dispersion have hitherto been unnoticed, and they correct Aristotle’s geography, pointing out that there are indeed two Pharos, one that is a small island off the coast of Alexandria (where the scorpions were, and still are, toxic), and the other that is also an island (Hvar, in the Adriatic Sea) that is inhabited by non-toxic scorpions.

### Elephants

Aristotle wrote about the elephant in several parts of the *History of Animals*, including its appetite,^62^ diseases,^63^ and use in war.^64^ However, there is some discussion about where these elephants came from and how Aristotle knew about them. For example, Romm (1989) argues strenuously against the common ancient belief that Aristotle’s knowledge of elephants was based on information he received from Alexander’s eastern conquests and that the only elephant that Aristotle knew was from India. Further, Romm argues that, perhaps due to Aristotle's detailed knowledge about elephants, he most probably received that information from an elephant handler rather than first-hand. However, Bigwood (1993) responds to Romm’s (1989) assertions by arguing that Aristotle was indeed talking about the Indian elephant and seemed to have no knowledge of the African species. Further, Bigwood (1993) claims that Aristotle’s knowledge of India was sparse and that he probably gained his information about Indian elephants from other sources, such as Ctesias, Eudoxus of Cnidus, and Callisthenes (who accompanied Alexander's Asian expedition). Nevertheless, Bigwood (1993) and Romm (1989) agree that “Aristotle's information about the elephant, scattered throughout the *Historia Animalium* and other biological works, is not only extraordinarily detailed but in most cases accurate as well” (Romm 1989, p. 537).

### Insects

Bodson (1983a, b) opens her discussion of Aristotle’s descriptions of insects by lamenting the relative lack of attention that most modern entomologists pay to Aristotle. Instead, she argues that modern entomology originated in ancient Greece and provides examples from Aristotle that include the sounds made by insects,^65^ how they sleep,^66^ hibernate,^67^ mate,^68^ and their reaction to being dissected.^69^

### Birds

In her paper addressed to comparative psychologists, Bodson (1996) provides a tabulated summary of sixteen aspects of bird breeding behavior, including forming pairs, nesting sites, courtship, egg laying, sitting period, hatching, and others. Bodson cross-references these aspects of bird breeding behavior across thirteen different species of birds, supported by twenty-two citations from Aristotle’s *History of Animals*.^70^

## Comment on the Selection of Topics by Modern Writers

Four aspects of the thirty-eight papers reviewed here are discussed below: their content, their publication outlet, the distribution of publications over the fifty-year period surveyed, and any trends in the topics studied in these publications. Table 1 presents a summary of those papers, grouped according to topic, and shows author(s), year published, and the source of material from Aristotle used in each paper.

It is apparent from Table 1 that papers were published on a range of topics, and that the most attention was shown to Aristotle’s descriptions of various creatures (13 papers), followed by those on his writing about anatomy (6 papers, or 7 papers if the writing on the brain is included). The paper by Clarke et al. (1963) was included because it gave a strong introduction to this work by Aristotle, even though it did not fall within the fifty-year period. Of the papers on creatures, by far the major focus is on Aristotle’s descriptions of fish and other sea creatures, although the contributions of Voultsiadou and her colleagues comprise the major portion of those papers. For instance, Voultsiadou and Vafidis (2007) list the 85 current marine invertebrate taxa that Aristotle accurately described, and Ganias et al. (2017) name 109 individual fish taxa mentioned by Aristotle. Not surprisingly, as well as the specific content (that is, animals, anatomy, etc.) of Aristotle’s work that these papers focus upon, the authors also share an admiration for Aristotle’s detailed and accurate observations. In dealing with the pointed criticisms made by Francis Bacon about Aristotle, Cosans (1998) does not hesitate to challenge Bacon’s comments by undertaking the same dissections with animals that Aristotle described.

Second, the selection of publication outlets also provides an insight into the goals of these authors who comment on Aristotle’s relevance to modern science. As shown in Table 1, the journals in which these papers appear cover a wide range of scientific fields. While there are four papers from the *Journal of the History of Biology*, there are also six papers from journals that include papers on the history of science (such as *History and Philosophy of the Life Sciences*, *Journal of the History of Ideas*, *Journal of the History of Medicine and Allied Sciences*, *Archives of Natural History,* and *American Journal of Philology*). Similarly, classics journals have also published some of these papers on Aristotle’s observations of animals (*Philosophical Transactions of the Royal Society*, *Acta Classica*, *Arethusa*, *The Classical Outlook*, and *Phronesis*). However, there are twenty publications in scientific journals that do not purport to discuss historical issues (*Journal of Evolutionary Biology*, *Perspectives on Science*, *Journal of Agricultural and Environmental Ethics*, *The Neuroscientist*, *Clinical Anatomy*, *Fish and Fisheries*, *Mediterranean Marine Science*, and others of the same genre). From a slightly different perspective, twenty-eight (or 73.7%) of the papers appear in science journals or journals devoted to the history of science, but only ten (or 26.3%) are published in history or classics journals. These data suggest that almost three-quarters of this selection of modern literature about Aristotle’s observation of animals was written for the specific consumption of scientists, while the remaining one-quarter could be intended to interest historians or classicists. On the basis that all these papers were peer-reviewed, it may be claimed that there is a reasonably active interest shown by scientists, historians, and classicists in Aristotle’s observations of animals.

**Table 1:** Summary details for peer-reviewed papers on Aristotle’s biology, 1970–2019, that report on mainstream biological topics

Topics	Authors (year)	Outlet	Aristotle source^b^
Side comments			
Genetics	Grene (2000)	*Perspectives on Science*	HA
Lifespans	De Magalhaes et al. (2009)	*J. Evolutionary Biology*	OLSL
Descriptions, reasoning	Brussow (2009)	*Philosophical Transactions of RS*^a^	BA
Anatomy	Auer et al. (2007)	*BMC Musculoskeletal Disorders*	BA
Gecko	Autumn et al. (2014)	*Ann Rev Ecol & Evol Systems*	HA
General papers			
Animal classification systems	Von Lieven et al. (2008)	*History & Philosophy of Life Sciences*	HA
Biological system structure	Grene (1972)	*J. History of Ideas*	HA
Genus-species differentiation	Lennox (198)	*J. History of Biology*	PA
Triune Brain	Smith (2010)	*J. History of the Neurosciences*	OtS
Soul	Corcilius et al. (2013)	*Phronesis*	MA
Genetics	Vinci et al. (2005)	*J. History of Ideas*	PA
	Kullman (1991)	*Acta Classica*	GA
	Henry (2006)	*J. History of Biology*	GA
Population biology	Egerton (1975)	*Arethusa*	HA
Animal care, agriculture	Grumett (2019)	*J. Agricultural & Environmental Ethics*	HA
	Brock (2004)	*The Classical Quarterly*	GA
The brain	Gross (1995)	*The Neuroscientist*	PA
	Clarke et al. (1963)^c^	*J. History of Medicine & Allied Sciences*	PA
Anatomy	Blits (1999)	*The Anatomical Record*	PA
	Malomo et al. (2006)	*Int. J. Morphology*	PA
	Crivellato et al. (2007)	*Clinical Anatomy*	PA
	Shoja et al. (2008)	*Int. J. Cardiology*	HA
	Shaw (1972)	*J History of Biology*	HA, PA
	Cosans (1998)	*Biology & Philosophy*	HA
Movement	Frampton (1991)	*J. History of Biology*	MA
Particular creatures			
Fish, other sea animals	Tipton (2006)	*Perspectives in Biology and Medicine*	HA
	Tipton (2008)	*Archives of Natural History*	HA
	Bodson (198)	*J. History of Biology*	HA
	Buddington et al. (1986)	*Proc. National Acad Sciences*	PA
	Voultsiadou (2007)	*J. Marine Biological Assoc. of the UK*	HA
	Voultsiadou and Vafidis (2007)	*Contributions to Zoology*	HA
	Ganias et al. (2017)	*Fish & Fisheries*	PA
	Voultsiadou et al. (2007)	*Mediterranean Marine Science*	HA
Scorpions	Fet et al. (2009)	*Boletin Sociedad Entomol. Aragonesa*	HA
Elephants	Romm (1989)	*American J. Philology*	HA
	Bigwood (1993)	*American J. Philology*	HA
Insects	Bodson (1983a, b)	*The Classical Outlook*	HA
Birds	Bodson (1996)	*Int J. Comparative Psychology*	HA

^a^*RS* Royal Society

^b^*HA* History of Animals, *OLSL *on longevity and shortness of life, *BA *“books on animals” (unspecified), *PA* parts of animals, *OtS* on the soul, *MA* movement of animals, *GA* generation of animals

^c^ Although not published within the 50-year period, this publication is of considerable relevance to later papers

**Fig. 1:**
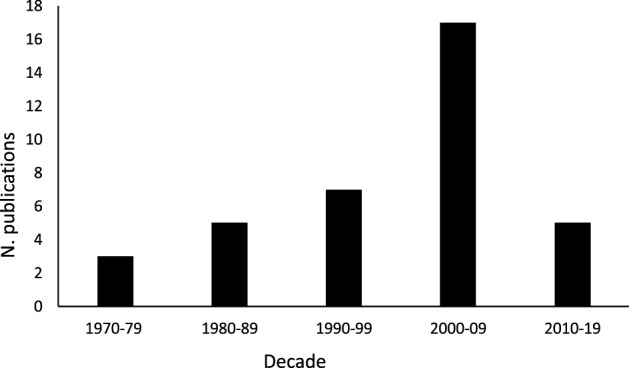
Publications per decade, 1970 to 2019

Third, it is also of interest to note the distribution over time of the papers identified for review. Figure 1 depicts that distribution on a decade-by-decade basis (excluding Clarke et al. 1963) and shows a fairly even appearance rate per decade except for the period from 2000 to 2009. No obvious explanation is forthcoming for this finding, and perusal of Table 1 indicates that none of the various topic areas is particularly present in this ten-year period; those seventeen papers that were published between 2000 and 2009 included comments on Aristotle’s worth as a scientist, his animal classification systems, genetics, animal care, anatomy, fish, and scorpions.

Finally, when the content and year are combined to detect any observable trends over time, as shown in Table 2, there is no obvious pattern according to the subjects of papers published over the five decades. Researchers appear to be consistently interested in Aristotle’s method of classifying species over this period, with papers describing this in the 1970s, 1980s, and 2000s. Similarly, anatomy retains its place in papers published in the 1970s, 1990s, and 2000s; and specific animals receive attention in each decade from the 1970s to the present. There are proportionally more papers on these topics according to the total number of publications in specific decades (the 2000s has many more of everything than the other decades), but no clear change in focus.

**Table 2:** Publication focus by decade published

Decade	1970–1979	1980–1989	1990–1999	2000–2009	2010–2019
Topics	Biological systems Population biology Anatomy	Genus-species differentiation Fish, other sea animals (2) Elephants Insects	Genetics Brain Anatomy (2) Animal movement Elephants Birds	Genetics (3) Lifespans Reasoning Anatomy (4) Animal classification Animal care Fish, sea animals (5) Scorpions	Gecko Brain Soul Animal care Fish

## Conclusion

While the relative status of Aristotle as a natural scientist vis-a-vis his traditional image as a philosopher has only relatively recently started to be debated in classical studies, his reception in modern science has steadily grown over the last five decades. Based on the papers reviewed here, plus the brief summary of their content, outlets, and timing of publication over a fifty-year period, it is argued that Aristotle’s observations of animals not only continue to excite interest in modern scientists but remain of relevance to modern biology and contribute to the general history of biology itself. Evidence of this claim is the extent to which highly-competitive publication space in mainstream historical, classics, and scientific journals is being given to papers that highlight Aristotle’s exact and voluminous output in what is now termed “biology,” his ability to demonstrate the principles of precise observation and hypothesis-formulation, and his fluid and informative communication with his audience. The fact that each of the 38 papers described herein was peer-reviewed by other researchers in the general fields of history, classics, and biology, and judged by them to be not only of relevance but also of sufficient scientific standing as indicators of an ancient but enormously comprehensive and still informative piece of work, further affirms the relevance of Aristotle’s work to modern science. As a statement about the ongoing history of biology itself, these papers also argue for the worth of an accumulative set of observations and deductions in the general field of biology, even from very long ago.

## Data Availability

Not applicable.

## Footnotes

1 Despite Cuvier’s staunch opposition to concepts of evolution as they were defined before Darwin’s *Origin of Species*, Darwin still admired Cuvier (Gotthelf 1999).

2 For the purpose of this paper, this will mostly be restricted to Aristotle’s *History of Animals, Parts of Animals, Movement of Animals, Progression of Animals,* and *Generation of Animals* (Aristotle 1984a, b, c, d, e), as sourced from Barnes (1984). The Greek texts of these works are critically available in the edited *Collection Budé* and *Oxford Classical Texts* series.

3 These will most often appear in mainstream scientific journals, but not always. See Table 1 for a list of outlets, including both historical and biological journals.

4 Google Scholar [GS] was chosen as the initial search engine so as to maximize the breadth of the search, then ratified by PubMed and Science Direct. This was reported by Martín-Martín et al. (2018), who note: “GS citation data is essentially a superset of WoS [Web of Science] and Scopus, with substantial extra coverage” (p. 1160.) Thus, GS was the first stage in identifying relevant papers, which were then reduced by checking for peer review.

5 Over 93% of these were not directly concerned with biology per se, but rather with other aspects of Aristotle’s writings in politics, philosophy, and ethics, or with philosophical aspects of his works on biology.

6 The search was restricted to peer-reviewed journal articles checked via each journal’s webpage self-description of editorial processes (rather than, say, books or comments in non-peer-reviewed outlets) to ensure that the quality of the papers was high, and that the purpose of the publication was to inform peer readers. Due to the sorting by relevance, the number of papers that could be used in this review decreased as the total number of articles identified by the search descriptors mounted. For the first 200 references to peer-reviewed articles, 26 relevant papers were identified; for the next 200 references, 4 relevant papers were identified; and for the final 100 references, no relevant papers were found. It is reasonable to assume from these data that the first 500 references identified by this Google Scholar search contained most, or all, of the relevant papers available for this review from that source. Supplementary searching in PubMed and Science Direct confirmed these findings.

7 Almost all were philosophical in focus, reflecting the relative weighting of papers overall. Many were also in book form, which were not included for the reasons of requiring a peer-review process inherent in academic journals.

8 No additional citations were found in PubMed, and only a few commentary papers appear in Science Direct, which have been cited herein.

9 For example: “The differences of birds compared one with another are differences of magnitude, and of the greater or smaller development of parts” (Aristotle, *Parts of Animals*, IV, 692^b^ 4–5).

10 For Aristotle, these were vegetative, sensation, and rational thought (Aristotle, *On the Soul*, II, 414a 30–31), and “the soul is inseparable from its body” (II, 413^a^, 4).

11 As is the argument put forward by Marcos that Aristotle’s biology is the key to the rest of his work; see Marcos (2012); on why Aristotle considered women to be inferior to men, see Lange 1983.

12 For example, “Aristotle's most significant achievement as natural scientist lies in his establishment of biology as an independent discipline” (Kullman 1991, p. 138).

13 “Aristotle succeeded in grouping animals according to the degree of their complexity” (Kullman 1991, p. 140).

14 “Here Aristotle's genetics are far superior to, for example, Darwinian genetics” (Kullman 1991, p. 145).

15 Aristotle actually observed that semen “is to be of such a nature that from it as their origin come into being those things which are naturally formed, not because there is any agent which makes them from it as … [missing text] … but simply because this is the semen” (Aristotle, *Generation of Animals*, I, 724^a^ 19–20).

16 “The same causes must be held responsible for the facts that some children resemble their parents, while others do not (some being like the father and others like the mother)” (Aristotle, *Generation of Animals*, IV, 767^a^ 38–39).

17 “the movement imparted by the male will make the form of the embryo in the likeness of itself” (Aristotle, *Generation of Animals*, IV, 767^b^ 16–17).

18 Aristotle, *Generation of Animals*, IV, 768^a^ 1.

19 Aristotle devoted the entire chapter 20 of *History of Animals,* Book VI, to describing the life cycle of the Laconian hound from conception (“The bitch conceives with one lining” [574^a^ 20]), through pregnancy (“The Laconian bitch carries her young the sixth part of a year” [574^a^ 23]), birth (“the pups are blind for twelve days after birth” [574^a^ 25]), maturation (“The male as a rule lifts his leg to void urine when six months old” [574^b^ 20]), lifespan (“The dog of the Laconian breed lives ten years, and the bitch twelve” [574^b^ 31]).

20 Probably from his exposure to his father’s medical guild practices, and also during his tutoring of Alexander and while at his Lyceum; see Shields (2012, p. 5).

21 For example, regarding cattle: “Cattle feed on fruit and grass, and fatten on vegetables that tend to cause flatulency, such as bitter vetch or bruised beans or bean-stalks:” Aristotle, *History of Animals*, VIII, 595^b^ 6–24, and followed by chapters on horses, elephants, and sheep.

22 Which “resides in a kind of origin of the body” (of animals) (Aristotle, *Movement of Animals*, I, 703^a^ 36 – 703^b^2).

23 Although this was the subject of a great deal of discussion in its own right (for example, see Gotthelf 2012), in this context, it may be accepted to loosely refer to "’that for the sake of which’ a series of events takes place is the intrinsic endpoint in which, if nothing fatally interferes” (Grene 1972, p. 397).

24 For example: “Shepherds train sheep to close in together at a clap of their hands, for if, when a thunderstorm comes, a ewe stays behind without closing in, it will miscarry if it be with young” (Aristotle*, History of Animals*, IX, 610b 35–36).

25 Aristotle, *Generation of Animals*, I, 730^a^ 6–7.

26 Brock’s footnote 1, attributed to A. Platt 1910, but no page number is given.

27 Aristotle, *Parts of Animals*, II, 656^a^ 24–25.

28 Aristotle, *Parts of Animals*, II, 656^a^ 28–29.

29 It has been suggested that this Museum was “a continuation and expansion of Aristotle’s school, the Lyceum”; see Gross (1995, p. 249).

30 Including the proximity to the eyes, and “because the direction in which sensation acts is in front” (Aristotle, *Parts of Animals*, II, 656^b^ 23–24).

31 “The brain lies in the front part of the head” (Aristotle, *History of Animals*, I, 494^b^ 25).

32 Including the proximity to the eyes, and “because the direction in which sensation acts is in front” (Aristotle, *Parts of Animals*, I, 993–1086; II, 656^b^ 23–24).

33 “The brain lies in the front part of the head” (Aristotle, *History of Animals*, I, 494^b^ 25).

34 “For the inner parts of man are to a very great extent unknown, and the consequence is that we must have recourse to an examination of the inner parts of other animals whose nature in any way resembles that of man” (Aristotle, *History of Animals*, I, 494^b^ 21–24).

35 Described as “an experimental means” and which consisted of comparing a series of eggs by opening them day by day to see the change in development of the embryo (Lennox 2012, p. 297).

36 “As every instrument and every bodily member is for the sake of something, viz. some action, so the whole body must evidently be for the sake of some complex action" (Aristotle, *Parts of Animals*, I, 645^b^ 15–16).

37 Aristotle, *Parts of Animals*, I, 645^a^ 23–24.

38 “For there is an upper and a lower half, a front and a rear, a right side and a left” (Aristotle, *Parts of Animals*, III, 669^b^ 21–21).

39 “Credit must be given to observations rather than to theories, and to theories only if what they affirm agrees with the observed facts” (Aristotle, *Generation of Animals*, III, 760^b^ 31–33).

40 Harvey also maintained Aristotle’s notion that “The heart of animals is the foundation of their life, the sovereign of everything within them, the sun of their microcosm, that upon which all growth depends, from which all power proceeds” (Harvey 1941, p. 3).

41 “In the largest animals all three chambers are distinctly seen” (see Aristotle, *History of Animals*, III, 513^a^ 30).

42 “Just as though the cavity were a part of the vessel, in which the blood forms a lake” (Aristotle, *History of Animals,* III, 513^b^ 5).

43 Defending it from Francis Bacon’s comment that Aristotle “utterly enslaved his natural philosophy to his logic” (Bacon 1994, p. 54).

44 “Desire” includes wish, impulse and appetite which “has been set going on the occasion of sensation or imagination”) because an animal’s limbs may become larger by virtue of becoming warmer as a result of “imaginations, sensations and by ideas” (Aristotle, *Movement of Animals*, I, 701^a^ 5–6).

45 Aristotle, *Movement of Animals*, I, 703^a^ 2.

46 Aristotle, *Parts of Animals*, II, 656^b^ 15. Aristotle noted that the sensory nerves for the eyes led into the brain rather than the heart: “Now since this origin is for some animals in the heart…for this reason the connatural spirit is there too” (Aristotle, *Movement of Animals*, 703^a^ 15).

47 Mentioned across several sites in Aristotle’s *History of Animals*, including VIII, 598^a^ 9, VIII, 601^b^ 17, IX, 621^b^ 13, VIII, 591^b^ 10 (“Other fish feed habitually on the mud or sea-weed or sea-moss or the so-called stalk-weed or growing plants as for instance, the phucis, the goby, and the rock-fish” (VI, 567^b^ 20).

48 Via “monthly sampling between September 2000 and August 2001” and again “in March 2003”; see Tipton (2006, p. 372).

49 Aristotle, *History of Animals*, VIII, 591^b^ 20.

50 Aristotle, *History of Animals*, VIII, 591^b^ 21.

51 He devotes two chapters to this in *History of Animals*: VI, Chapters 10, 11.

52 Aristotle, *History of Animals*, II, 508^b^ 16.

53 Aristotle, *Parts of Animals*, III, 675^a^ 13–14.

54 Aristotle refers to these animals in several sections of *The History of Animals*, such as V, 548^b^, VIII, 588^b^21, I, 487^b^9 (“many creatures live in close adhesion to an external object, as is the case with several kinds of shellfish” [V, 548^a^29]); (“In chambers cavities of sponges pinna-guards are found. And over the chambers there is a kind of spider’s web, by the opening and closing of which they catch minute fishes” [V, 548^b^16]).

55 These are what Aristotle refers to as ‘animals devoid of blood” (*anhaima*): *History of Animal*s, IV, 523^b^ 1–2.

56 “The skin is in some fishes covered with scales…. In others it is rough…. Fewest of all are those whose skin is smooth” (Aristotle, *Parts of Animals*, IV, 697^a^ 5–8).

57 Here he made one of his major errors by claiming that some fish reproduced spontaneously from mud: “Eels are derived from the so-called ‘earth’s guts’ that grow spontaneously in mud and humid ground” (Aristotle, *History of Animals*, VI, 570^a^, 16–17).

58 “Fish living near the shore are better eating than deep-sea fish” (Aristotle, *History of Animals*, VIII, 598^a^ 2).

59 “This fish swims northwards into the Ister, and then at the point of its bifurcation swims down into the Adriatic” (Aristotle, *History of Animals*, VIII, 598^b^ 15–16).

60 “The coly-mackerel is caught more frequently on entering than on quitting the Euxine” (Aristotle, *History of Animals,* VIII, 598^b^ 27).

61 Aristotle, *History of Animals*, VIII, 607^a^ 15–17.

62 “It can take six or seven medimni (of barley), five medimni of wheat, and five mareis of wine” (Aristotle, *History of Animals,* VIII, 596.^a^ 5–7). (A medimni was about 55 L.)

63 Including “flatulence,” diarrhoea,” and “insomnia” (Aristotle, *History of Animals*, VIII, 605^a^ 24–30).

64 “Indians employ these animals for war purposes” (Aristotle, *History of Animals*, IX, 610^a^ 19).

65 “Some of them make a humming noise … others are said to sing” (Aristotle, *History of Animals*, IV, 535^a^5-6).

66 “It is plain that insects sleep; for there can be no mistaking their condition of motionless repose:” Aristotle, *History of Animals*, IV, 537^b^ 5–6.

67 “Insects almost all go into hiding” (Aristotle, *History of Animals*, VIII, 599^a^ 20).

68 “Insects copulate at the hinder end, and the smaller individuals mount the larger; and the smaller individual is the male” (Aristotle, *History of Animals*, V, 542^a^ 1).

69 “All insects when cut in two continue to live” (Aristotle, *History of Animals*, IV, 531^b^30).

70 For example, regarding nesting: “Birds of a heavy build, such as quails, partridges, and the like, build no nests; indeed, here they are capable of flight, it would be no use if they could do so” (Aristotle, *History of Animals,* IX, 613^b^ 5–7). Similarly, about pigeon’s hatching behaviour: “The male takes its turn of sitting during the daytime; the female sits during the night” (Aristotle, *History of Animals*, VI, 562^b^ 17–18).
